# BCR-ABL1 Tyrosine Kinase Complex Signaling Transduction: Challenges to Overcome Resistance in Chronic Myeloid Leukemia

**DOI:** 10.3390/pharmaceutics14010215

**Published:** 2022-01-17

**Authors:** Gustavo P. Amarante-Mendes, Aamir Rana, Tarcila Santos Datoguia, Nelson Hamerschlak, Gabriela Brumatti

**Affiliations:** 1 Departamento de Imunologia, Instituto de Ciências Biomédicas, Universidade de São Paulo, São Paulo 05508-000, Brazil; aamirrana_grg@yahoo.com; 2 Instituto de Investigação em Imunologia, Instituto Nacional de Ciência e Tecnologia (INCT), São Paulo 05508-000, Brazil; 3 Department of Immuno-Oncology, Beckman Research Institute of City of Hope, Duarte, CA 91010, USA; 4 Hospital Israelita Albert Einstein, São Paulo 05652-900, Brazil; tarcila.datoguia@einstein.br (T.S.D.); hamer@einstein.br (N.H.); 5 Inflammation Division, Walter and Eliza Hall Institute of Medical Research, Melbourne, VIC 3052, Australia; brumatti@wehi.edu.au; 6 Department of Medical Biology, University of Melbourne, Melbourne, VIC 3010, Australia

**Keywords:** BCR-ABL1, chronic myeloid leukemia, tyrosine kinase inhibitors

## Abstract

The constitutively active BCR-ABL1 tyrosine kinase, found in t(9;22)(q34;q11) chromosomal translocation-derived leukemia, initiates an extremely complex signaling transduction cascade that induces a strong state of resistance to chemotherapy. Targeted therapies based on tyrosine kinase inhibitors (TKIs), such as imatinib, dasatinib, nilotinib, bosutinib, and ponatinib, have revolutionized the treatment of BCR-ABL1-driven leukemia, particularly chronic myeloid leukemia (CML). However, TKIs do not cure CML patients, as some develop TKI resistance and the majority relapse upon withdrawal from treatment. Importantly, although BCR-ABL1 tyrosine kinase is necessary to initiate and establish the malignant phenotype of Ph-related leukemia, in the later advanced phase of the disease, BCR-ABL1-independent mechanisms are also in place. Here, we present an overview of the signaling pathways initiated by BCR-ABL1 and discuss the major challenges regarding immunologic/pharmacologic combined therapies.

## 1. Introduction

**Figure 1:**
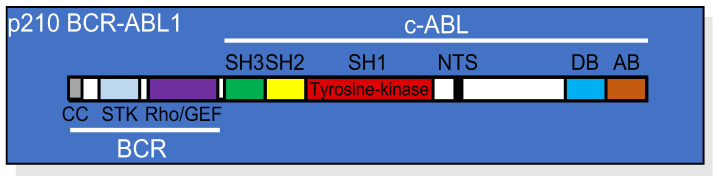
Linear structure of p210 BCR-ABL1 showing the relative position of each domain from both the BCR and the c-ABL portions of the protein. CC, coil-coiled oligomerization domain; STK, serine/threonine kinase domain; Rho/GEF domain; SH, (SRC homology domains) 1, 2, and 3; NTS, nuclear translocation signal; DB, DNA binding domain; AB, actin binding domain.

BCR-ABL1 is a multidomain, constitutively active, chimeric tyrosine kinase that results from a reciprocal translocation between chromosomes 9 and 22—t(9;22)(q34;q11)—characteristic of Philadelphia chromosome(Ph1)-positive leukemia [1]. Depending on the breakpoint on chromosome 22 at the *BCR* (break point cluster) gene, three major isoforms of BCR-ABL1 can be produced: the 185kDa, 210kDa, and 230kDa proteins found in acute lymphocytic leukemia (ALL), chronic myeloid leukemia (CML), and chronic neutrophilic leukemia (CNL), respectively [2,3,4]. In all these circumstances, the first exon of *c-ABL*—the cellular homolog of Abelson murine leukemia virus (A-MuLV)—on chromosome 9 is replaced by one of the *BCR* sequences (Figure 1).

The BCR part of the protein contributes to several domains responsible for regulating the enzymatic activity of BCR-ABL1 or its interactions with different binding partners [5,6]. At the N-terminal portion of BCR, there is a coiled-coil domain responsible for oligomerization and constitutive activation of the BCR-ABL1 tyrosine kinase. In addition, the BCR sequence contains a serine/threonine kinase (STK) domain, a Ras homolog gene family/guanine nucleotide exchange factors (Rho/GEF) kinase domain, and SH2 domains capable of binding adaptor molecules, such as growth factor receptor-bound protein 2 (GRB2) [7].

c-ABL is a tyrosine kinase located preferentially in the nucleus [8], although it is also found in the cytoplasm where it associates with actin filaments [5]. The structure of c-ABL, which is conserved in BCR-ABL1, comprises multiple domains, including SRC-homology domain 1 (SH1; kinase domain), SH2, SH3, DNA binding (DB), and actin binding (AB) domains, in addition to a nuclear translocation signal (NTS) sequence, sites for phosphorylation by protein kinase C (PKC), and a proline-rich sequence (Figure 1). Among all these domains, the SH1 region is the most conserved during evolution and contains the catalytic site essential for the initiation of signaling pathways that result in cellular transformation, including dysregulated proliferation and resistance to apoptosis.

The indication that this enzymatic activity was essential to induce the transformation of BCR-ABL1-positive cells led to the development of a rationally designed tyrosine kinase inhibitor (TKI)—imatinib mesylate, also known as STI571 or Gleevec [9]. Imatinib was the first TKI anticancer targeted therapy to receive FDA approval and has unquestionably changed the outcomes of a great number of CML patients. This TKI has prolonged the overall survival of BCR-ABL positive leukemia patients to the point that their life spans are now similar to age-matched healthy individuals [10]. However, with time, approximately 50% of patients develop resistance or intolerance to imatinib and treatment must be discontinued. Second generation TKIs (e.g., dasatinib, nilotinib, and bosutinib) exhibit significantly improved activity against all resistant mutations except BCR-ABLT315I, affecting threonine 315 which is crucial for the accessibility of the ATP-binding pocket (see below), and have been used as either salvage therapies or alternative first-line treatments [11]. Finally, ponatinib, a high potency third generation TKI, has shown unique activity against the BCR-ABLT315I mutation; however, it has also been associated with considerable risks for vascular occlusions, heart failure, and hepatotoxicity [12,13]. Despite the significant improvement of the treatment of BCR-ABL1-drived leukemia, some patients still develop intolerance or resistance to all TKIs, progress to a more advanced phase of the disease, and/or require continuous resistance to TKI therapy. Importantly, at this point, kinase-independent or even BCR-ABL1-independent signals may trigger alternative survival pathways responsible for the residual presence of leukemic cells in these patients.

## 2. BCR-ABL1 Tyrosine Kinase-Dependent Signaling Cascade

Due to its complex structural nature, multiple proteins have been shown to directly associate with BCR-ABL1. Co-immunoprecipitation of BCR-ABL1 from the K562 CML cell line followed by mass spectrometry revealed the potential core components of the BCR-ABL1-interactome, including the adapter proteins GRB2, SHC adaptor protein 1 (SHC1), CT10 regulator of kinase 1 (CRK1), the E3 ubiquitin-protein ligase casitas B-lineage lymphoma (c-CBL), the p85α and β subunits of the phosphoinositide 3-kinase (PI3K), the suppressor of T-cell receptor signaling 1 (STS1), and the SH2 domain-containing inositol 5-phosphatase 2 (SHIP-2) [14]. These proteins, along with other interactors, can recruit intermediate and/or effector molecules, thereby initiating a plethora of signaling pathways, including RAS/RAF/MAPK, PI3K/AKT/mTOR, JAK/STAT, and WNT/β-catenin, briefly presented below, responsible for the different aspects of BCR-ABL-1-induced transformation.

### 2.1. BCR-ABL1 Activation of the RAS/RAF/MAPK Pathway

The activation of the rat sarcoma virus (RAS)/rapidly accelerated fibrosarcoma (RAF)/mitogen-activated protein kinase (MAPK) pathway by BCR-ABL1 is initiated by GRB2 binding to phosphotyrosine Y177 in the BCR region, followed by the recruitment of GRB2-associated-binding protein 2 (GAB-2) and son of sevenless (SOS). The BCR–ABL1–GRB2–SOS complex drives RAS activation and the consequent activation of RAF1, MAPK/ERK Kinase (MEK), extracellular signal-regulated kinase (ERK), c-Jun N-terminal kinase (JNK), and p38MAPK. Together, these signaling pathways regulate cell proliferation, differentiation, and survival [15,16,17,18]. Most importantly, expression of a dominant-negative RAS inhibits BCR-ABL1-mediated transformation and attenuates CML-like myeloproliferative disease [19]. It is important to note that RAS can also contribute to the activation of the pro-survival PI3K/AKT/mTOR pathway [20].

### 2.2. BCR-ABL1 Activation of the PI3K/AKT/mTOR Pathway

BCR-ABL1 activates the PI3K/AKT/mTOR pathway both directly and indirectly through the induction of autocrine cytokines [21]. The interaction between BCR-ABL1 and PI3K can occur via GRB2, GAB-2, SHC, c-CBL, and CRKL [15,22,23]. BCR-ABL1 activates the p85 regulatory subunit of PI3K leading to the conversion of phosphatidylinositol 4,5-bisphosphate (PIP2) to phosphatidylinositol 3,4,5-trisphosphate (PIP3), which further activates phosphoinositide-dependent kinase 1 (PDK1) and AKT [24]. Activated AKT has been shown to prevent apoptosis by phosphorylating different substrates, such as glycogen synthase kinase 3 (GSK3), cysteine-aspartic protease-9 (caspase-9), and BCL-2 associated agonist of cell death (BAD) [25]. Importantly, BAD phosphorylation is not essential for BCR-ABL1-induced survival [26]. Although PI3K activation is implicated in the antiapoptotic effect induced by growth factors, such as NGF, PDGF, and IGF-1—but not IL-3 [27,28,29]—PI3K activity appears not to be involved in the resistance to chemotherapeutic drugs observed in BCR-ABL1-positive cells [30].

AKT also induces the downstream activation of mammalian target of rapamycin (mTOR), which works as a catalytic subunit of the mTORC1 and mTORC2 protein complexes [31]. mTORC1 activates eukaryotic translation initiation factor 4E-binding protein 1 (4EBP1), S6 Kinase (S6K), and S6 ribosomal protein (RPS6) to control cell cycle progression from the G1 to S phase, cell proliferation, and angiogenesis. On the other hand, mTORC2 regulates cytoskeleton organization and cell proliferation [32]. BCR-ABL1-mediated induction of the mTOR pathway also upregulates the FOXO subclass of forkhead box transcription factors (FOXO1, FOXO3a, and FOXO4) to promote leukemogenesis [33]. In leukemic stem cells (LSCs), dysregulated PI3K/AKT/mTOR signaling activates LKB1 and AMPK via ROS production to regulate cell survival, proliferation, and drug resistance [34,35].

Importantly, resistance to TKIs, such as imatinib, dasatinib, and nilotinib, is associated with the hyperactivation of the mTORC2/AKT signaling pathway [36,37], possibly via BCR-ABL1-independent PI3K activation [38]. Targeted inhibition of mTORC2/AKT signaling by glucocorticoid-induced leucine zipper protein (GILZ) dampens imatinib and dasatinib resistance and restricts tumor growth. Interestingly, the binding of GILZ to mTORC2, but not to mTORC1, reduces AKT phosphorylation (at Ser473) and induces FOXO3a-mediated transcription of the proapoptotic protein BCL-2-interacting mediator of cell death (BIM) [39]. Combined targeting of mTORC1 and mTORC2 by OSI-027, a catalytic mTOR inhibitor, also results in a strong anti-leukemic effect against BCR-ABL1-positive cells [40], which can be prevented by concomitant inhibition of autophagy [41]. Equally, the use of PI3K inhibitors LY294002 and CAL-10 restored apoptosis and sensitivity to TKI inhibitors in leukemic cells [42].

### 2.3. BCR-ABL1 Activation of the JAK/STAT Pathway

Janus kinase (JAK)/signal transducer and activator of transcription (STAT) signaling is involved in growth factor independence and resistance to apoptosis in CML [43,44,45,46,47]. JAK2, STAT1, STAT3, and STAT5 were shown to be constitutively active in CML cell lines [47]. BCR-ABL1-mediated activation of STAT5 regulates the transcription of BCL-2-related protein A1 (A1), B-cell lymphoma extra-large (BCL-xL), and myeloid cell leukemia-1 (MCL-1) all potent anti-apoptotic members of the BCL-2 family [48,49,50,51]. Importantly, the inhibition of JAK2 or STAT5 was shown to induce apoptosis in BCR-ABL1-positive cell lines as well as primary cells derived from imatinib-sensitive or resistant CML patients [45,46,52].

### 2.4. BCR-ABL1 Activation of the WNT/β-Catenin Pathway

A disturbance in the canonical WNT/β-catenin signaling pathway is associated with the pathogenesis of leukemia [53,54]. In the absence of WNT activation, β-catenin is phosphorylated and ubiquitinated in the context of a multiprotein complex composed of glycogen synthase kinase 3 (GSK-3), creatin kinase 1 (CK1), axis inhibitor (Axin), adenomatous polyposis coli (APC), protein phosphatase 2A (PP2A), and the E3-ubiquitin ligase β-transducin repeat-containing protein (β-TrCP), followed by further proteasomal degradation [53,54]. BCR-ABL1 directly binds and phosphorylates β-catenin [55], resulting in its stabilization and nuclear translocation/accumulation [56], which has been implicated in the proliferation and survival of leukemic stem cells in CML patients [17]. In the nucleus, β-catenin interacts with members of the T cell factor/lymphoid enhancer factor (TCF/LEF) family of transcription factors (TCF1, TCF3-4, and LEF1) to activate target gene expression, including c-MYC and cyclin D1 [57]. Importantly, genetic or pharmacological inhibition of β-catenin were shown to interfere with CML cell proliferation and induce apoptosis, including in cells bearing the T315I mutation [58].

### 2.5. BCR-ABL1 Activation of the PP2A Pathway

PP2A is a tumor suppressor serine-threonine phosphatase that negatively regulates the mitogenic and survival signals emanating from PI3K/AKT, RAS/MAPK, and MYC pathways [59]. Interestingly, PP2A is downmodulated by BCR-ABL1 in CML patients, particularly during blast crisis [60,61]. BCR-ABL1 inhibition of PP2A is mediated by the activation of SET, an endogenous inhibitor of PP2A. Importantly, treatment with imatinib, inhibition of SET, or pharmacological activation of PP2A lead to inactivation and degradation of BCR-ABL1 and, consequently, the loss of tumorigenic activity in BCR-ABL1-positive cells, including TKI-resistant CML stem cells [62]. At least in part, the inhibitory effect of PP2A is mediated by the activation of another tumor suppressor phosphatase, namely Src homology region 2 domain-containing phosphatase-1 (SHP-1) [60,61], as well as the inhibition of JAK2 and β-catenin [62].

## 3. BCR-ABL1 Kinase-Independent Alternative Survival Signals

Unquestionably, the BCR-ABL1 tyrosine kinase-dependent signaling events summarized above are required for the transformation of Ph1 chromosome-positive leukemia. However, the resistance to TKIs observed in some CML patients suggests that signals emanating from the BCR-ABL1 protein independently of its tyrosine kinase activity may take over, allowing the survival of leukemic cells and relapse of the disease. Indeed, it was recently shown that inhibition of the catalytic activity does not completely dismantle the BCR-ABL1 molecular complex [63]. Signaling proteins, such as p85α-PI3K, GRB2, SHIP2, SHC1, SOS1, and c-CBL, remain associated with BCR-ABL1, whereas CRK, CRKL, or GAB2 seem to detach from the complex [63]. Therefore, residual signaling transduction events appear to be sufficient to maintain survival of CML cells in the absence of tyrosine kinase activity, as previously proposed [64]. This idea is supported, for instance, by the observation of the alternative activation of RAF/MEK/ERK, mTOR, or PKC-β/Alox5 pathways in imatinib-resistant patients, which could be circumvented using TKI + mTOR or PKC combined therapies [65,66,67].

Equally important is the fact that LSCs homing to bone marrow niches is arguably the most important event related to TKI resistance, since it results in a high genetic, epigenetic, and transcriptional alteration leading to an increased self-renewal capacity of the LSCs themselves [61]. Acquired heterogeneity helps some LSCs to survive TKI treatment, thus serving as a reservoir of leukemic cells that can eventually cause relapse after therapy discontinuation [68]. Furthermore, microenvironment alterations at the hematopoietic niche may contribute to the survival of LSCs. Altered metabolic pathways and the expression of co-stimulatory molecules and/or regulatory cytokines by surrounding stromal cells may create an immunosuppressive environment that helps malignant cells to escape immune surveillance and targeted chemotherapy [69,70].

## 4. Resistance to Apoptosis in CML

As discussed above, BCR-ABL1 activates multiple signaling pathways to induce leukemogenesis, which results in growth factor-independency and regulation of adhesion and invasion [71,72,73]. On the other hand, BCR-ABL1-positive cells generally display normal mitotic indices and do not show increased overall proliferation [74]. Perhaps the most notable aspect of BCR-ABL1-mediated leukemogenesis is the vigorous state of resistance to apoptosis that is conferred to the transformed cells [19,75,76,77,78,79,80].

The enforced expression of BCR-ABL1 in hematopoietic cell lineages revealed its potential to prevent apoptosis induced by a variety of stimuli, including growth factor withdrawal, γ-irradiation, death receptor agonists, and multiple chemotherapeutic drugs [19,75,76,77,78,79]. Studies with point mutations at the autophosphorylation site (Y793F), the phosphotyrosine binding motif (R552L), and/or at the GRB2-binding site (Y177F) demonstrated that BCR-ABL1-mediated resistance to apoptosis depends on the cellular context. For instance, enforced expression of a BCR-ABL1-Y177F/R552L/Y793F triple mutant in IL-3-dependent lymphoblastoid 32D murine cells did not confer IL-3 independency or resistance to γ-irradiation-induced apoptosis; however, the same mutant protected BaF3 cells, a different IL-3-dependent pro-B murine cell line, from these apoptogenic stimuli [19]. Moreover, enforced expression of wild type or the above-mentioned BCR-ABL1 mutants protected the apoptosis-sensitive human acute promyelocytic leukemia HL-60 cell line from a variety of apoptogenic insults to the same extent [77]. Therefore, different cellular contexts may provide alternative pathways that contribute to the survival of BCR-ABL1-positive cells.

BCR-ABL1 has been shown to inhibit apoptosis at the mitochondrial level by preventing the release of cytochrome c to the cytosol and the consequent activation of effector caspases [77]. It is important to note that cytochrome c release is controlled by members of the BCL-2 family of proteins [81,82]. Interestingly, BCR-ABL1 expression has been associated with the downregulation of BCL-2 and upregulation of A1, BCL-xL, and MCL-1 [30,50,51,83], which are all anti-apoptotic proteins critical to the regulation of mitochondria-mediated cell death. The upregulation of MCL-1 seems to involve the activation of the RAS/RAF/MAPK pathway [51] and Sphingosine kinase-1 (SPK1) [84]. While increased expression of A1 and BCL-xL depends on BCR-ABL1 tyrosine kinase activation of STAT5, and the inhibition of STAT5, BCL-xL, or MCL-1 renders BCR-ABL1-positive cells susceptible to apoptosis [48,49,51,77]. In addition, there is a positive correlation between STAT5 activity, BCL-xL levels, and the progression of disease in CML patients [85]. Nevertheless, BCL-xL is only partially responsible for the BCR-ABL1-mediated resistance to apoptosis [77]. In agreement with this notion, a systematic comparison of the ectopic expression of either BCR-ABL1, BCL-xL, or BCL-2 in HL-60 cells revealed a remarkably robust anti-apoptotic effect conferred by BCR-ABL1, greater than the resistance awarded by either of the anti-apoptotic proteins of the BCL-2 family [79], reinforcing the idea that BCR-ABL1 acts on multiple points of the apoptosis machinery.

The regulation of particular pro-apoptotic BH3-only members of the BCL-2 family were also shown to contribute to the resistance to apoptosis observed in BCR-ABL1-positive cells. As previously mentioned, BCR-ABL uses the PI3K pathway to phosphorylate and inactivate BAD; however, as mentioned previously, BAD inactivation does not account for the resistance to apoptosis observed in BCR-ABL1-positive cells [26]. Interestingly, imatinib was shown to kill BCR-ABL1-positive cells via modulation of members of the BCL-2 family [86]. BIM and BAD are post-translationally activated by imatinib, and the elimination of both confers resistance to imatinib [86]. More importantly, a common intronic deletion polymorphism of *BIM* that produces BIM isoforms lacking the pro-apoptotic BH3 domain has been associated with resistance to TKIs in CML [87]. Apparently, other members of the BH3-only subfamily of BCL-2 proteins, such as BID, BIK, BMF, HRK, NOXA, and PUMA, the anti-apoptotic protein BCL-w, as well as the apoptotic pore-form proteins BAX and BAK, do not seem to be modulated by BCR-ABL1 or play an important role in BCR-ABL1-mediated leukemogenesis.

BCR-ABL1 has also been shown to inhibit apoptosis initiated by death receptor signaling [77,79,80,88,89,90]. Tumor necrosis factor-related apoptosis-inducing ligand (TRAIL/TNFSF10), which was shown to induce apoptosis in many tumor cells but not in normal cells [91,92,93], is downregulated in CML patients, particularly in more advanced phases of the disease [90,94]. The downregulation of TRAIL in BCR-ABL1-positive cells is mediated by a preferentially expressed antigen of melanoma (PRAME)/enhancer of zeste homolog 2 (EZH2) repression mechanism, and the inhibition of either PRAME or EZH2 expression increases TRAIL levels and sensitivity to TRAIL and to imatinib [90]. Finally, BCR-ABL1 was additionally shown to inhibit apoptosis downstream of cytochrome c release from mitochondria by interfering with APAF-1-caspase-9 apoptosome activation [95].

## 5. Tyrosine Kinase Inhibitors and the Paradigm Shift of CML Treatment

**Figure 2:**
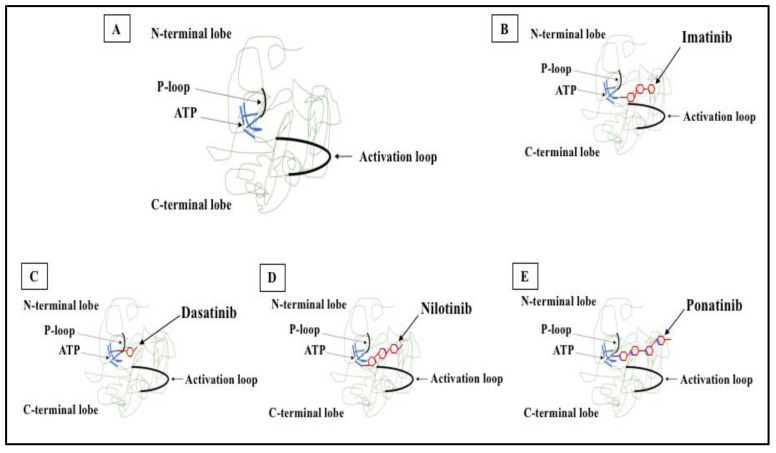
2D structure of BCR-ABL1 kinase domain and binding sites for TKIs. (**A**) BCR-ABL1 is a constitutively active kinase that binds ATP and transfers a phosphate from ATP to tyrosine residues on various substrates. This activates downstream signaling pathways, leading to abnormal cellular adhesion and proliferation of myeloid cells and inhibition of apoptosis. TKIs were developed to specifically block the binding of ATP to the BCR-ABL tyrosine kinase, inactivating the constitutive tyrosine kinase activity and inhibiting downstream pathways. (**B**) Imatinib (first generation TKI) binds to the BCR-ABL kinase domain in its inactive conformation through the ATP binding site. (**C**) Dasatinib (second generation TKI) inhibits the BCR-ABL tyrosine kinase performance at the ATP site in ABL regardless of protein conformation (active or inactive). (**D**) Nilotinib (second generation TKI) connects to an inactive conformation of the BCR-ABL protein, taking an analogous region that would be occupied by ATP. (**E**) Ponatinib (third generation TKI) has multiple contact points for the inactive conformation of the ABL and for the T315I mutation.

The discovery that constitutive BCR-ABL1 tyrosine kinase activity was crucial for the development of CML [96] warranted a TK-targeting therapeutic strategy. Consequently, several TKIs were developed to target the ATP binding site of the kinase domain, thereby preventing phosphorylation of the target protein and subsequent signaling events (Figure 2). Imatinib, nilotinib, dasatinib, bosutinib, ponatinib, and asciminib are currently used for the treatment of CML and are briefly described below.

### 5.1. Imatinib Mesylate

Imatinib was developed by Novartis Pharmaceuticals and was the first TKI to successfully show an impact on the proliferation and survival of BCR-ABL1-expressing cells in vitro and in vivo [9,97]. In 2001, imatinib was FDA approved for the treatment of three clinical stages of CML: CML-BC (blast crisis); CML-AP (accelerated phase); and CML-CP (chronic disease resistant or intolerant to interferon-alpha (IFN-α) treatment). The approval of imatinib transformed the treatment of CML patients. Early results from phase III randomized studies showed that imatinib was more effective than the standard-of-care combined therapy, cytarabine and interferon-alpha (IFN-α), for the treatment of patients with newly diagnosed CML. At 18 months, the estimated rate of complete cytogenetic response was around 76% in imatinib-treated patients, compared with 14.5% in patients receiving IFN-α/cytarabine [98]. Further studies revealed a survival advantage for imatinib first-line treatment over IFN-α/cytarabine combination therapy [99]. The promising low side effects and high efficiency of imatinib were further confirmed in a ten-year follow up study, with an overall survival rate of 83% [100]. Despite the low toxicity and high efficiency of this therapy, approximately 25% of the patients eventually developed resistance to imatinib, while 5–10% discontinued the therapy due to intolerance [101]. Resistance to imatinib is often associated with mutations in the BCR-ABL1 kinase domain that allow evasion of TKI binding and consequently the reactivation of BCR-ABL1 oncogenic signal transduction. However, alternative resistance mechanisms have also been found in patients with full inhibition of BCR-ABL1 [102]. This led to the development of second generation TKIs.

### 5.2. Nilotinib

The TKI nilotinib was FDA approved in 2007 for the treatment of CML-AP and -CP patients who were resistant or intolerant to imatinib treatment [103]. In vitro studies showed that nilotinib was 20 to 50-fold more potent than imatinib, exhibiting inhibitory activity against the majority of BCR-ABL1 imatinib-associated mutations, except for the T315I mutation [104]. A phase III randomized study showed that, at 12 months, nilotinib was more efficient than imatinib for the treatment of patients with newly diagnosed CP-BCR-ABL positive CML, where the complete cytogenetic response of nilotinib-treated patients was 80%, compared to 65% for imatinib [105]. Although nilotinib reduced the occurrence of treatment-associated BCR-ABL1 mutations when compared to imatinib [106,107], four kinase domain mutations were less sensitive to this TKI treatment. The imatinib-associated mutation T315I remains as a marker for resistance to nilotinib. Additionally, nilotinib was also less effective at eliminating leukemia cells from patients presenting mutations in the amino acids F359C/V, E255K/V, and Y253H [102]. Interestingly, 61% of the patients on nilotinib treatment that progressed to advanced phases of the disease had no newly detectable mutations, suggesting alternative mechanisms of resistance in these patients [108].

### 5.3. Dasatinib

Dasatinib is a potent second-generation inhibitor targeting several kinases, including BCR-ABL1, SRC kinases, c-KIT, and platelet-derived growth factor receptor beta (PDGFR-β) [109,110]. In vitro and in vivo studies have shown that dasatinib inhibits proliferation of BCR-ABL1-positive cells and prolongs survival in mouse models induced by imatinib-resistant cells [111]. However, dasatinib is less effective for the elimination of T315I-positive leukemia cells. Clinical studies have shown that the treatment of imatinib-resistant or intolerant chronic CML patients induced a 43% molecular response [112], whereas patients who switched to dasatinib after 3 months of imatinib treatment had a significantly higher molecular response than patients who remained on imatinib [113]. This result suggests that second generation TKIs may provide clinical benefit for CML patients as a second line of treatment, after 3 months of treatment with imatinib, with a possible reduction in resistance.

### 5.4. Bosutinib

Bosutinib is a second-generation TKI with dual inhibitory activity against SRC and ABL kinases. Different than dasatinib, this inhibitor has minimal inhibitory effects against c-KIT and PDGFR-β kinases [114,115]. In Phase I/II clinical trials, bosutinib was effective against imatinib-resistant and intolerant leukemias. However, despite all efforts to create a more potent inhibitor, as with other TKIs, bosutinib was less effective for the treatment of CML with T315I mutations [116,117]. Compared to nilotinib and dasatinib, bosutinib has a very similar efficacy in the treatment of imatinib-resistant CML. The advantage of this inhibitor is a higher safety profile. Transient mild-to-moderate myelosuppression was observed in the early days of treatment, with neutropenia/thrombocytopenia of 18% and 24%, respectively, for bosutinib patients, compared to 33–49% and 22–47% for dasatinib and 13–29% and 20–29% for nilotinib. Furthermore, hemorrhagic events have been observed less often in bosutinib treated patients (5%) compared to other TKI inhibitors (40% for dasatinib) [116]. These results may be associated with a lack of activity of bosutinib towards the inhibition of c-KIT and platelet function, important for normal hematopoiesis and blood coagulation, respectively [118,119]. Thus, bosutinib may provide a potent molecular response with less side effects for imatinib-resistant CML patients.

### 5.5. Ponatinib

The third-generation TKI ponatinib is a BCR-ABL1 inhibitor with strong activity against common ABL kinase domain mutations, including the T315I mutation [120]. In vitro and in vivo studies have shown that ponatinib inhibits the catalytic activity of native and mutant ABL, thereby interfering with proliferation and inducing apoptosis in cells expressing BCR-ABL1 mutants, as well as prolonging the survival of mice with BCR-ABL T315I-dependent disease [120]. Due to its promising effect against other BCR-ABL1 mutations, in 2012, ponatinib was granted an accelerated FDA approval for the treatment of CML patients resistant to imatinib. In phase II trials with this drug, 34% of CML patients had a molecular response. Of those, 56% presented the T315I mutation. Moreover, no single mutation conferring resistance to this TKI was observed [121]. These results suggest a strong efficiency of ponatinib for the elimination of BCR-ABL1 leukemic cells. However, serious arterial thrombotic events were observed in 9% of the patients receiving ponatinib [122,123]. The severity of the side-effect raised concerned from the US FDA and the inhibitor had to be withdrawn from the market. In 2014, the drug was back on the market with a safer treatment regimen and clear restrictions of its use regarding vascular conditions [124].

### 5.6. Asciminib

Asciminib (ABL001/Sclembix) is the newest tyrosine kinase inhibitor that targets both native and mutated BCR-ABL1, including the gatekeeper T3151 mutant. Different to other TKIs, asciminib does not bind to the ATP-binding site of the kinase portion of BCR-ABL, but rather works as an allosteric inhibitor of kinase activity. Asciminib binds to the myristoyl site of the kinase domain, normally occupied by a motif that serves as an allosteric negative regulatory element. Through binding of this site, asciminib locks BCR-ABL into an inactive conformation inhibiting downstream signaling events [125,126]. In October 2021, the US Food and Drug Administration (FDA) granted accelerated approval of asciminib for the treatment of CML in two specific indications: (i) for adult patients with Philadelphia chromosome-positive CML in the chronic phase (Ph+ CML-CP) with a low molecular response rate (MMR) at 24 weeks post-treatment with two or more TKIs and (ii) for adult patients with Ph+ CML-CP with the T315I mutation. The initial results revealed that asciminib had a cytogenetic response rate of 41%, compared to 24% for the TKI bosutinib, indicating the potential value of asciminib therapy for TKI-resistant patients.

Although TKIs have offered a significantly improved survival and quality of life for patients with CML, the ability of this agent to eradicate quiescent CML stem cells remains a challenge. New therapies that inhibit leukemia stem cells and consequently reduce the chances of relapse are currently under investigation.

## 6. Mechanisms of Resistance to TKI

**Figure 3:**
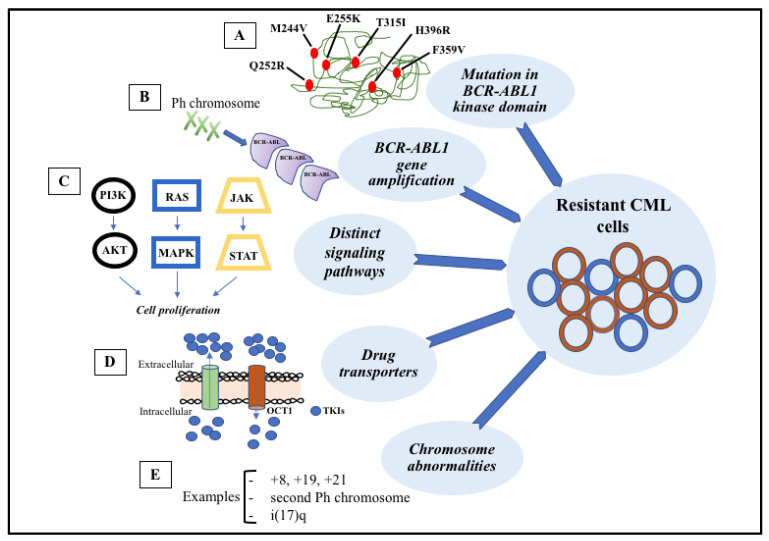
Signaling pathways involved in the development of target therapy resistance. (**A**) Molecular structure of BCR-ABL1 kinase domain with some mutations (indicated in red). (**B**) Gene amplification can lead to overproduction of tyrosine kinase. (**C**) Constitutive activation of signaling pathways, such as PI3K-AKT, RAS-MAPK, and JAK-STAT, result in cell proliferation and anti-apoptotic mechanisms. (**D**) Intracellular concentrations of TKIs can be modified through membrane transporters that may cause increased efflux or decreased influx. (**E**) The most common chromosome abnormalities involved in karyotype evolution are trisomy 8, trisomy 19, trisomy 21, second Ph chromosome, and isochromosome 17.

The major purpose of CML therapy with TKIs is to achieve a deep molecular response. To identify those who have not achieved desired response and may be suffering from resistance to TKIs, it is necessary to apply the European LeukemiaNet criteria for treatment response [124,127]. It is possible to rate the success of therapy by monitoring the BCR-ABL1 transcript levels at 3, 6, and 12 months, and any time after 12 months (Table 1). To maintain the current treatment, an optimal response is mandatory; otherwise, the therapy must be replaced (failure/resistance) or considered for change (warning) [124]. Furthermore, to achieve successful treatment, it is necessary to understand the mechanisms that led to the poor outcome, particularly the resistance to TKIs (Figure 3).

Resistance to TKIs can be classified as primary (no hematologic or cytogenetic response from the beginning of therapy) or secondary (initial response that decays during the treatment). Currently, two mechanisms of resistance are known: BCR-ABL1-dependent and BCR-ABL1-independent pathways [122]. It is important to note that the ATP molecule binds between the two lobes of the catalytic domain in ABL kinase (an N-terminal lobe and a C-terminal lobe) [128]. Mutations that lead to imatinib resistance have been detected in the phosphate-binding loop and in other regions of the kinase domain where amino acid substitutions may result in conformational changes that prevent imatinib binding [129].

The first report of TKI resistance was published in 2001 when a group of patients lost response during imatinib treatment [130]. The sequencing of the BCR-ABL1 kinase domain in these patients revealed a single nucleotide change at position 315 (T315I) in six of nine cases. Importantly, the threonine at position 315 (Thr315) is the “gatekeeper” that controls the accessibility of the ATP-binding pocket [131]. When replaced by an isoleucine (T315I), patients develop pan-resistance to TKIs: it prevents the drug from attaching to the ATP-binding pocket (for example, dasatinib) or impairs the drug from binding to kinase due to a conformational change from the inactive type II conformation to active type I (for instance, imatinib and nilotinib).

**Figure 4:**
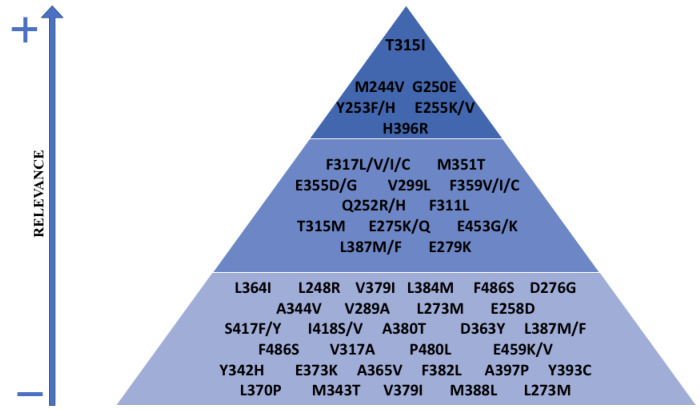
List of the most frequent *BCR-ABL1* kinase domain mutations resistant to ATP-competitive inhibitors according to their relative relevance.

Several mutations were described, some more frequent than others, for example, T315I, H396P, E255K, Y253F [132], and mostly for amino acid substitutions at the kinase domain [128] (Figure 4). Interestingly, some mutations are strongly associated with disease stages, e.g., substitutions at H396, L248, and F317 in chronic phase and T315, E255, and Q252 in accelerated phase, and others with ethnicity [133].

Amplification or overexpression of the ABL1 kinase domain triggered by genetic instability can also impair TKI binding and consequently reactivation of the phosphorylation cascade [134]. Although the amplification rate is higher per cell division, clinically, it is more common to find point mutations, likely because overexpression of BCR-ABL1 provokes cell injury. As BCR-ABL1 transcript levels correlate with the disease phase, high levels in the advanced phase may be related to the development of resistance [135]. Indeed, a study confirmed this finding, showing a high level of BCR-ABL1 transcripts during the resistance period [23]. Another important aspect is clonal evolution, defined by the acquisition of secondary abnormalities leading to chromosomal aberration. These abnormalities can exist already at diagnosis or develop during treatment of CML and are mainly associated with disease progression (transformation to accelerated or blastic phase) [136]. According to European LeukemiaNet, the “major route” abnormalities are trisomy 8, second chromosome Ph, isochromosome 17, and trisomy 19 [137,138,139]. The relationship between the appearance of clonal evolution and decreased survival is well described in the literature [140]. One study described 171 patients who did not achieve a complete response (at the beginning of the disease), and clonal evolution was present at the time of imatinib failure. In this group of patients, BCR-ABL1-kinase mutations occurred more with clonal evolution than those without [134].

Resistance to TKI therapy can occur independently of BCR-ABL1 and may involve genomic instability, epigenetic modification, the overexpression of multidrug resistance (MDR) proteins involved in the efflux of anti-cancer drugs, the activation of survival pathways (Ras/MAPK, JAK/STAT, PI3K/AKT), and interactions with the LSC microenvironment [141].

The genomic instability of LSCs contributes to the emergence of genetic abnormalities. Mutations in the coding sequences of genes involved in chromatin remodeling, differentiation, proliferation, and survival of blood cells, including ASLX1, RUNX1, and NRAS, may contribute to progression and drug resistance in CML [142,143]. In addition, it is well known that epigenetic dysregulation plays an important role in the maintenance and progression of cancer. There is growing evidence that epigenetic events may also have a role in TKI resistance [144,145,146].

The ATP-binding cassette (ABC) transporter family of proteins actively exports structurally unrelated substrates out of cells, presumably to protect them from possible toxicities. Overexpression of some members of this family, including P-glycoprotein (MDR1) and breast cancer resistance protein (ABCG2), have been implicated in leukemia stem cell drug resistance [70,147,148,149]. CML patients in blast crisis present with higher expression of MDR1 compared to CML patients in the chronic phase, and MDR1 upregulation has been associated with decreased sensitivity to chemotherapy in advanced-phase disease [147,150]. Inhibitors of MDR1 and ABCG2 are available and have passed phase I and II clinical trials’ safety requirements. It is possible that combined treatment with these drugs may increase the sensitivity of CML-LSCs to TKIs and improve prognosis for treatment relapse patients. Organic cation transporters (OCTs) are known to affect substrate transport, and some studies have demonstrated that imatinib enters cells through OCT-1. With OCT inhibition, the levels of imatinib were also decreased in cells, explaining this dose-dependent relation but not clinical resistance [148]. These studies developed several important hypotheses and possible paths to improve combined therapy.

As discussed above, BCR-ABL1 induces the activation of multiple signaling pathways, such as JAK-STAT, RAS-MAPK, and PI3K-AKT, that are responsible for cell proliferation and anti-apoptotic signaling [11]. Although treatment with TKIs somehow disables these pathways, they can be reactivated by alternative and external pathways, making the tumor cell independent of BCR-ABL1, and promoting resistance to therapy [151,152]. For instance, BCR-ABL1-independent activation of STAT3 was found in TKI-resistant cell lines and primary CML cells from patients with clinical resistance to multiple TKIs [153], suggesting that STAT3 activation is a critical survival pathway in CML.

Several recent studies have shown that the bone marrow microenvironment plays an important role on the response of leukemic cells to anti-cancer drugs. CML stromal cells have an abnormal gene expression pattern, despite a deep molecular response, that may contribute to disease relapse and secondary resistance [154]. These cells may promote cell cycle arrest in LSCs through specific signals, thus allowing their persistence during TKI treatment [155]. Expression of CXCL12 MSCs, but not other CXCL12-expressing BM microenvironment cell populations, has been shown to be important for the persistence of quiescent, TKI-resistant LSCs within the BM microenvironment [156]. In addition, stromal cell-derived cytokines, such as granulocyte-macrophage colony-stimulating factor (GM-CSF), fibroblast growth factor-2 (FGF-2), and placental growth factor (PIGF), promote BCR-ABL1-independent proliferation and resistance to cell death [157,158,159].

Finally, an important element to be considered is the pharmacokinetics of TKIs. A study showed that imatinib concentrations may vary due to the levels of cytochrome p450 isoenzyme 4A, which is responsible for neutralizing it, directly impairing the response to treatment [160].

## 7. Challenges and Future Perspectives—Novel Agents and Combined Therapies

Despite the extensive knowledge about molecular resistance to TKIs, the biology involved in BCR-ABL1-independent mechanisms needs better elucidation. Specific molecules are under investigation as possible and potential therapeutic targets. As described previously, the activation of signaling pathways, such as PI3K-AKT, can lead to TKI resistance. The phosphorylation of FOXO transcription factors that occurs through the AKT pathway can also take place across BCR-ABL1, leading to changes in cell cycle regulation related to proliferation, differentiation, and cell death [161]. In vitro assays have shown that treatment with PI3K inhibitors can elevate cytoplasmic FOXO levels and increase patients’ response to more than one TKI [162].

Another pathway that should be better analyzed is WNT-β-catenin signaling. This pathway is associated with a variety of proliferative diseases, including CML [163]. WNT-β-catenin signaling is involved in several aspects of CML, such as stem cell maintenance, the self-renewal capacity of myeloid blastic phase-CML, and CML persistence in murine disease models through the activation of β-catenin [164]. Therefore, targeting nuclear β-catenin should be considered in patients who do not respond to TKIs in the absence of BCR-ABL1 mutations.

An alternative option for a therapy target is AXL, a tyrosine kinase receptor found to be overexpressed in imatinib- and nilotinib-resistant CML cell lines and patients, as well as associated with AKT survival signaling [165,166]. Downregulation of AXL was shown to partially reverse imatinib or nilotinib resistance [165,166], as well as the self-renewal capacity of primary BCR-ABL1-positive CD34+ stem cells [167]. Finally, treatment with an AXL inhibitor reduced tumor growth in mice inoculated with BCR-ABL1 T315I-bearing CML cells [168].

Another field that should be explored is oxidative stress. Some studies demonstrate that BCR-ABL1 increases the number of reactive oxygen species (ROS) in the cell, promoting mutations through oxidative damage to DNA. Antioxidant therapies that lead to ROS inhibition decrease mutagenesis and, consequently, TKI resistance [169].

Vascular endothelial growth factor receptor (VEGFR) also plays a role in CML, and the use of VEGFR inhibitors has been shown to lead to a decrease in resistance to TKIs [170]. An example is axitinib—when linked to ABL1, it can inhibit BCR-ABL1 in the presence of the T315I mutation [171].

BH3 mimetics, small molecule inhibitors of the prosurvival members of the Bcl-2 family, have been developed for the treatment of cancer and, particularly, hematological malignancies [172,173,174]. Venetoclax/ABT-199/ GDC-0199 is the first selective inhibitor of BCL-2 to show clinically relevant antitumor activity without causing thrombocytopenia [175]. Venetoclax was shown to sensitize BIM-deficient TKI-resistant cell lines and patient samples to imatinib [86,87]. Venetoclax in combination with imatinib or nilotinib was able to disrupt the engraftment potential of CML precursors, possibly by reducing their ability to form colonies and/or inducing apoptosis [176,177]. More importantly, a retrospective study in CML and Ph+ AML patients.

Finally, in a different avenue, strategies aiming to degrade the BCR-ABL1 protein using proteolysis-targeting chimera (PROTAC) technology have been tested. PROTACs are small molecules with a heterobifunctional structure—part of the molecule consists of a specific ligand to the protein of interest (POI) linked to an E3 ligase-recruiting domain [178,179,180,181,182]. The approximation of the target protein to the E3 ligase results in ubiquitination and degradation of the former by the proteasome [178,179,180,181,182]. Importantly, BCR-ABL1-targeted PROTACs were shown to inhibit proliferation and induce apoptosis in cell lines and in primary CML CD34+ cells [183,184,185].

It can be emphasized that within malignant hematological diseases, an exceptional result in the overall survival of patients with CML was achieved through the development of TKIs. There are obstacles related to treatment management, including the appearance of mutations and a loss of response after discontinuing TKIs. Despite these problems, the results show increased treatment effectiveness and manageable side effects compared to those obtained in other chronic leukemias with a high risk of progression to an acute form. Researchers are on the right path to improve the knowledge already consolidated and seek to understand alternative therapies that can either function synergistically with existing treatments or are superior to current therapies. Notwithstanding, efforts are still in place to find better BCR-ABL1 kinase inhibitors that can overcome multiple TKI-resistant mutants [186,187,188].

## Figures and Tables

**Table 1:** Criteria for optimal, warning, and failure response to any TKI as first-line therapy.

Time	Optimal	Warning	Failure
Baseline	NA	High risk ACA, high risk ELTS score	NA
3 months	BCR-ABL1 ≤10%	BCR-ABL1 >10%	BCR-ABL1 >10% if confirmed within 1–3 months
6 months	BCR-ABL1 ≤1%	BCR-ABL1 >1–10%	BCR-ABL1 >10%
12 months	BCR-ABL1 ≤0.1%	BCR-ABL1 >0.1–1%	BCR-ABL1 >1%
Any time	BCR-ABL1 ≤0.1%	>0.1%; loss of ≤0.1% (MMR) *	BCR-ABL1 >1%, resistance mutations high risk ACA

* Loss of MMR (BCR-ABL1 > 0.1%) means failure after treatment remission. NA, not applicable; ELTS, EUTOS score; ACA, additional chromosomal aberrations; MMR, major molecular remission.

## Data Availability

Not applicable.
